# Patient safety problems from healthcare information technology in medical imaging

**DOI:** 10.1259/bjrcr.20150107

**Published:** 2015-12-16

**Authors:** Timothy J Schultz, Natalie Hannaford, Catherine Mandel

**Affiliations:** ^1^ Australian Patient Safety Foundation, South Australian Health and Medical Research Institute (SAHMRI), Adelaide, SA, Australia; ^2^ School of Psychology, Social Work and Social Policy, University of South Australia, Adelaide, SA, Australia; ^3^ Brain and Psychological Sciences Centre, Faculty of Health, Arts and Design, Swinburne University of Technology, Melbourne, VIC, Australia; ^4^ Department of Radiology, University of Melbourne, Melbourne, VIC, Australia

## Abstract

Health information technology (HIT) systems have been deployed extensively by healthcare organizations and promoted as a panacea to many of the challenges faced by medical imaging departments, particularly with respect to workflow, efficiency and diagnostic accuracy. This report describes how inadequate planning, integration, training and testing of HIT can impact on patient safety and result in patient harm.

## Introduction

Health information technology (HIT) has been defined as:

“hardware or software that is used to electronically create, maintain, analyse, store, receive (information), or otherwise aid in the diagnosis, cure, mitigation, treatment or prevention of disease and that it is not an integral part of (1) an implantable device or (2) medical equipment”.^1^

HIT systems, particularly those used in radiology such as clinical decision support, picture archiving systems (PACS) and radiology information systems, are commonly promoted as the solution to many of the challenges faced by radiology departments.^2^ Radiologists, radiographers and referrers now rely on them to aid the diagnosis and treatment of patients. While there is some evidence for the clinical and operational benefits of such systems,^3^ there is growing evidence that PACS and similar systems are capable of harming patients.^4^

This case (Box 1) is a HIT incident recently reported into the Radiology Events Register (RaER; www.raer.org.au). This is a voluntary, anonymous, radiology-led incident reporting system in use in Australia and New Zealand that was developed to inform quality improvement initiatives in radiology.^5,6^ Other avenues exist for clinician-reported information about patient safety, including hospital-based reporting systems, and external regulatory or legal agencies whose primary roles are to ensure public accountability.^7^ However, anonymous and blame-free reporting using a quick and easy system that provides feedback and can lead to system improvement will maximize opportunities to learn from adverse events and “near misses”.^8,9^ Identifying information collected by RaER is protected from disclosure under statutory immunity legislation.^6^ However, even in optimal reporting systems, the barriers to incident reporting are significant, particularly for doctors.^10^ Enhancing engagement with doctors, targeted incident reporting, and effective triaging and robust analysis are essential for the success of incident reporting system’s *raison d’être* of improving the safety of healthcare.^11^ Although incident reporting systems are a good way of detecting safety issues and trends and alerting others to issues, they cannot, as non-mandatory systems, provide reliable information on the incidence of events.^7^

Box 1. The information technology incident reported to the Radiology Events Register database by an anonymous user showing responses to the main categories of information collected by the reporting system. As this is an anonymous report, it is not possible to ask further questions about the event.

**Description of incident:** An upgrade to the picture archiving systems (PACS) took longer than expected and resulted in the loss of functionality in the PACS. The next day it was evident that the images could not be transferred to PACS patient folders. This resulted in delayed reporting of images that were acquired but not loaded into PACS.

**Contributing factors:** Unexpected difficulties with the upgrade and lack of situational awareness as to the severity or impact of this issue and no plan of action being available for dealing with this problem.

**Consequence/outcome:** Delayed reporting and referrers unable to view images in a timely manner.

**Action taken:** All radiologists were made aware of the problems and arrangements were made for the radiologists to work late to report the outstanding studies.

**Preventability:** Having plans for PACS upgrades with contingency and potential problem action plans and redundancy capability in place. Ensuring adequate communication of problems across departments, with escalation if problems are encountered and having PACS health dashboards, alerts and escalation policies and procedures.

**Reporter:** Radiologist.

## Discussion

Despite widespread and enthusiastic uptake in many quarters, a large review of the impact of HIT on patient safety in 2012 reported “mixed” evidence.^12^ A summary of very recent evidence, however, stated that the quality and safety of healthcare has been clearly improved by HIT, but

“research should now turn to understanding the relatively small but important number of unintended consequences…especially in areas that impact patient safety”.^13^

This finding supports a 2013 US poll of safety, quality improvement and healthcare administration leaders, which identified HIT safety as the hazard of greatest concern.^14^ The case presented here demonstrates how one HIT error can have numerous potential adverse outcomes for many patients and how a concerted effort is required to ensure the reliability of HIT systems to acquire, store and support radiologist interpretation and reporting of images and the distribution of reports. Although the outcomes reported in the case were not specific, other studies have provided examples of actual patient harm from similar errors. For example, in a study of 46 HIT events in which patients were harmed, computerized physician order entry and PACS were involved in 93% of cases.^15^ In one instance, a patient died when a network problem in the PACS delayed transmission of images to a remote site for diagnosis, and in another example, the failure of a system to produce reports following a software upgrade was associated with a patient missing out on a liver transplant.^15^

Medical practitioners are given minimal training on the use and operation of HIT systems (*personal observations*), reflecting a wider lack of appropriate skills assessment and training in HIT skills in the healthcare setting.^16^ Training should therefore be built into any HIT vendor contract, and protected radiologist time for initial training should be built into the radiologist’s schedule.^17^ Learning curves will vary between users—for example, part-time staff may require more training, and ongoing systems training may be required for new radiologists or to update skills and after major upgrades.^17^ Large departments should plan for decreased productivity during rollout of a new system, and should consider employing full-time on-site vendor application specialist or providing in-house training.^17^ Developing radiologist and radiographer “super-users” with specialist knowledge who will share this knowledge with others should also be considered.^16,17^ The lack of training can result in less ability to use the technology optimally and decreased satisfaction on the part of the users who may struggle with the systems.

System flaws or issues identified with HIT updates or integration should be addressed by appropriate personnel in a timely manner using principles of system resilience and high reliability organizations.^9^ Creating resilient systems means ensuring that the same types of errors are not repeated, and HITs must be redesigned to reduce our dependence on vigilant practitioners and auditing processes to detect and prevent patient harm.^4^ Reliable systems to identify and fix HIT problems decrease healthcare practitioners’ stress and dissatisfaction, especially if the errors increase the risk of harming patients.

One of the primary goals of a PACS system is to increase the availability and timeliness of results at multiple locations to numerous providers and referrers. This goal can be severely hindered when data availability errors or “crashes” occur. These can be from unexpected downtime owing to an upgrade as in the case study presented, interoperability failures, lack of access to electronic medical records, failures related to being able to access previous radiology reports and studies,^2^ and integration or firewall issues. All of them can result in delayed diagnosis and treatment and potential harm to patients, and increased risks of litigation. As shown in the case, radiologists were required to work late to address the problem, and such ameliorating actions could conceivably impact on their satisfaction in the workplace. We could find no published research on the recommended frequency of upgrades to PACS.

Based on a systems approach to human error,^9^ PACS-specific data availability errors^4^ and an emerging evidence base on HIT impacts on patient safety in medical imaging,^15–18^ we propose the following 11 strategies that may be beneficial as a starting point in minimizing the risk of patient harm from problems with radiology HIT (Box 2). The 11 proposed strategies are grouped into five key areas related to: (i) preparation and integration of new systems; (ii) staff training and updates; (iii) appropriate information technology (IT) support; (iv) contingency planning to deal with problems; and (v) appropriate configuration and communication between systems. These factors should be considered at the time of design and purchase of systems, and should be supported by the availability of well-trained, dedicated IT staff to manage HIT systems. The ubiquity of HIT in current radiology practice demands a greater focus from all stakeholders, in particular: radiologists; practice, quality and/or departmental managers; accreditors; and professional organizations who set the standards of practice. For example, what constitutes: appropriate backup or emergency/contingency planning measures, acceptable downtime in outpatient and inpatient practice, and capacity for printing films. Similar to much of HIT, the evidence base to guide recommended practice in these areas is still developing. The need for guidance for the radiology profession is urgent.

Box 2. Strategies to reduce and manage the risk of health information technology (HIT) error.

*Preparation and integration for new and upgraded systems*

- Work with vendors and your organization to ensure appropriate integration of new HITs in your facility.
- Harmonize terminology, exposure indicators and information systems when products or versions from multiple vendors are being used.
- Carefully schedule HIT system changes to minimize disruption to normal workload and ensure contingency plans are in place (see below).

*Training for radiologists and radiographers*

- Train staff prior to the integration of HITs.
- Provide staff with training updates as a refresher and following system upgrades.

*Appropriate information technology (IT) support*

- Ensure timely access to designated IT personnel who are sufficiently trained in all aspects of the HITs in use in your facility.

*Contingency planning*

- Have detailed plans and emergency operation modes for managing unexpected downtimes.
- Effectively communicate planned or unplanned outages to all staff and referrers.
- Have adequate escalation procedures in place to deal with problems with the potential to cause patient harm.

*Configuration and communication between systems*

- Configure HITs (picture archiving systems/radiology information systems/electronic referral or computerized physician order entry) so that they are interoperable and communicate with each other.
- Ensure access to prior studies—pre-fetching algorithms and display protocols.

## Conclusions

Data availability errors can occur as a result of inadequate planning, unexpected downtime without adequate backup, lack of foresight and, importantly, poor understanding of integrating HIT systems into a practice. The general strategies suggested above aim to reduce and manage the risk of HIT error. Ensuring that individual settings protocols for managing planned and unexpected downtime of HIT systems are fit for purpose and robust is warranted. As there are a multitude of settings, technologies and healthcare systems potentially affected, it is difficult to specify in further detail; however, further discussion about general HIT issues in medical imaging is a good place to start.

Capturing and analyzing radiology incident data helps to engage doctors in patient safety, identify errors specific to radiology, work out how they occur and provides an opportunity to develop and communicate potential preventative and corrective strategies.
